# Artificial Intelligence in Cardiac Surgery and Surgical Training: Opportunities, Risks, and Safeguards for Preserving Expertise

**DOI:** 10.3390/jcm15166313

**Published:** 2026-08-15

**Authors:** Lazar Velicki, Aleksandra Milovancev, Andrej Preveden, Jelena Vuckovic, Miodrag Belopavlovic, Milan Rodic, Nenad Filipovic, Djordje Jakovljevic

**Affiliations:** 1 Faculty of Medicine, University of Novi Sad, 21000 Novi Sad, Serbia; aleksandra.milovancev@imf.uns.ac.rs (A.M.); andrej.preveden@mf.uns.ac.rs (A.P.); jelena.vuckovic@mf.uns.ac.rs (J.V.); miodrag.belopavlovic@ikvbv.ns.ac.rs (M.B.); milan.rodic@ikvbv.ns.ac.rs (M.R.); 2 Institute of Cardiovascular Diseases Vojvodina, 21208 Sremska Kamenica, Serbia; 3 Bioengineering Research and Development Center (BioIRC), 34000 Kragujevac, Serbia; fica@kg.ac.rs; 4 Faculty of Mechanical Engineering, University of Kragujevac, 36000 Kragujevac, Serbia; 5 Translational and Clinical Research Institute, Faculty of Medical Sciences, Newcastle University, Newcastle upon Tyne NE2 4HH, UK; djordje.jakovljevic@newcastle.ac.uk; 6 Clinical Sciences and Translational Medicine Research Theme, Research Centre for Health and Life Sciences, Institute of Health and Wellbeing, Coventry University, Coventry CV1 5FB, UK

**Keywords:** artificial intelligence, cardiac surgery, machine learning, surgical education, automation bias, surgical skills, clinical governance

## Abstract

Artificial intelligence (AI) is entering cardiac surgery through predictive modelling, multimodal imaging, perioperative monitoring, workflow automation, and emerging computer-vision applications. The most mature evidence concerns risk prediction before and after surgery. Even in this domain, however, systematic reviews show that improvements over conventional statistical models are often modest and that routine clinical implementation remains limited. In surgical education, simulation, automated video analysis, and objective performance metrics may expand opportunities for deliberate practice and provide feedback that is less dependent on individual observers. Most of this evidence comes from general, laparoscopic, urological, and robotic surgery rather than cardiac-specific training, and its transferability should not be assumed. The same technologies also create risks. Automation bias, cognitive off-loading, reduced exposure to failure management, and displacement of mentor–trainee interaction may weaken the independent judgement on which safe cardiac surgery depends. Opaque models, dataset shift, inequitable performance, and uncertain accountability add further clinical and ethical concerns. This narrative review examines the current and emerging roles of AI across the cardiac surgical pathway and in cardiothoracic training, while distinguishing demonstrated applications from plausible but unproven uses. We propose a human-in-command framework based on external validation, local performance testing, transparent intended use, preserved manual and crisis-management competencies, simulation of technology failure, faculty oversight, competency-based credentialing, and continuous audit. AI should be judged not by technical novelty alone but by whether it improves care while preserving the ability of surgeons and teams to operate safely when the technology is unavailable or wrong.

## 1. Introduction

Artificial intelligence (AI) has moved rapidly from a largely experimental field to a practical component of contemporary medicine. In broad terms, AI encompasses computational systems that perform tasks commonly associated with human intelligence, including pattern recognition, prediction, language processing, and decision support. Machine learning is a subset of AI in which models learn associations from data rather than relying exclusively on prespecified rules. Deep learning extends this approach through multilayer neural networks that can process complex inputs such as images, physiological signals, free text, and surgical video [1,2,3].

Cardiac surgery appears particularly suitable for these methods. The specialty generates large volumes of structured and unstructured data before, during, and after an operation: demographic and laboratory variables, electrocardiograms, echocardiographic and computed-tomographic images, perfusion and anaesthetic records, operative video, intensive care time series, and longitudinal outcomes. These data could support more individualized risk estimation, earlier recognition of deterioration, improved workflow, and more objective assessment of technical performance [4,5,6,7]. At the same time, cardiac surgery is an unforgiving environment. Decisions are time-sensitive, anatomy is variable, complications may evolve abruptly, and safe performance depends on tacit knowledge, team coordination, and the ability to improvise when the expected plan fails.

The central question is therefore not whether AI will enter cardiac surgery but how it should be integrated. Enthusiasm can obscure an important distinction between technical feasibility and clinical value. A model may achieve excellent discrimination in a retrospective dataset yet add little to an established risk score, fail after transfer to another institution, or provide information too late to change management. Similarly, an automated assessment system may classify expert and novice movements in a simulator without demonstrating that its feedback improves operative performance or patient outcomes. The educational consequences require equal scrutiny. AI may enhance access to practice and feedback, but excessive reliance on algorithmic guidance could weaken independent problem-solving, manual versatility, and the apprenticeship relationships through which surgical judgement is formed.

This review examines AI across the cardiac surgical pathway, with particular emphasis on surgical education and preservation of expertise. It deliberately separates established evidence from emerging applications and from claims that remain speculative. The aim is not to resist technological change but to define the conditions under which AI can improve safety and learning without displacing the human capabilities that remain essential to cardiac surgery.

## 2. Scope and Approach of This Narrative Review

**Figure 1:**
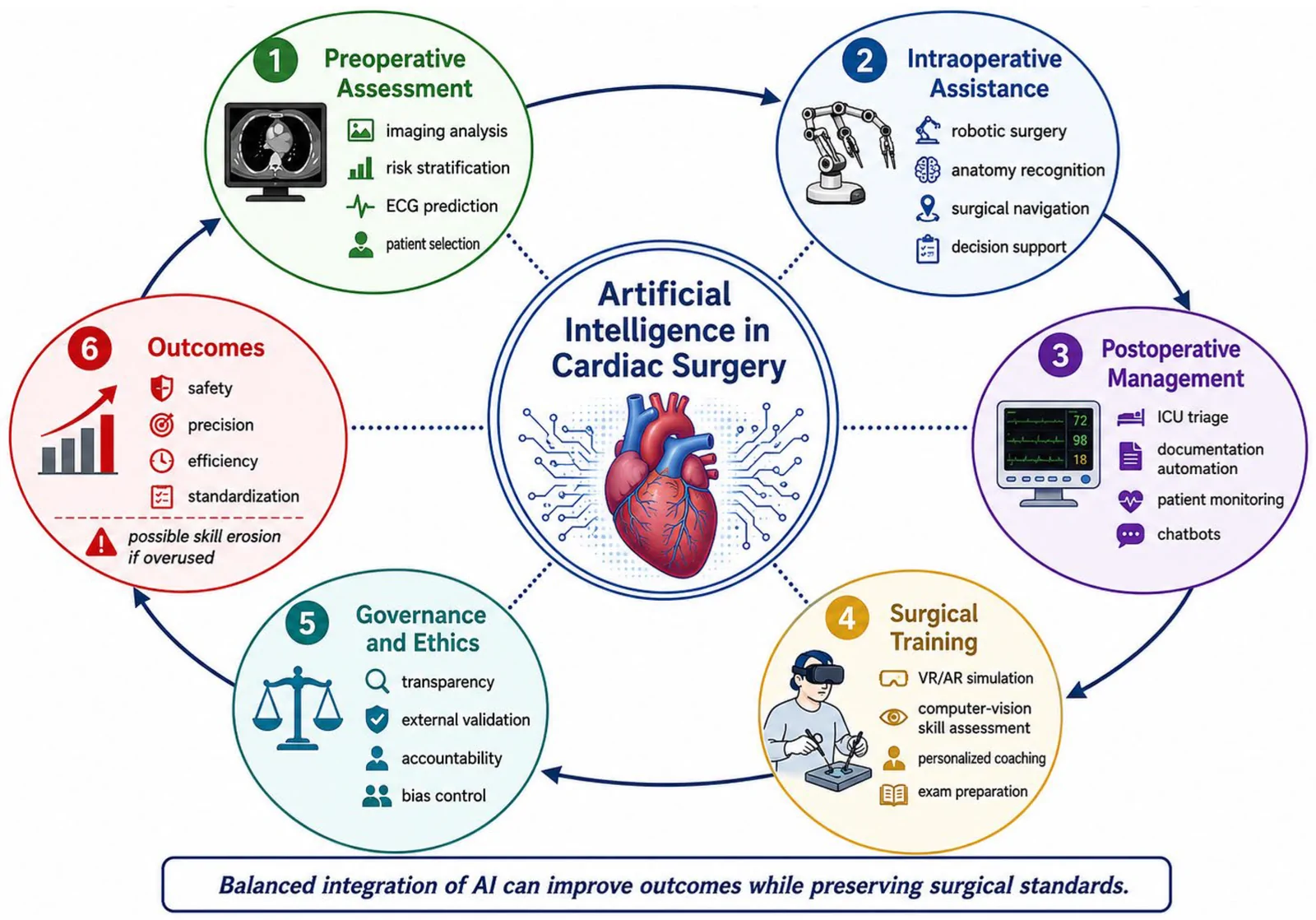
Principal domains of artificial intelligence application in cardiac surgery, including preoperative assessment, intraoperative assistance, postoperative management, surgical training, governance and ethics, and expected outcomes. The outer arrows indicate the linked clinical and learning pathways, while the radial connections show that data and decision support can operate across domains; governance and ethics apply throughout, and observed outcomes feed back into validation and monitoring. The figure is conceptual and does not imply equal evidence maturity: cardiac-specific evidence is most developed for risk prediction; imaging-supported planning and postoperative tools are emerging; and autonomous intraoperative guidance and most AI-enabled training applications remain investigational and often rely on evidence extrapolated from non-cardiac specialties. ECG, electrocardiography; ICU, intensive care unit; VR, virtual reality; AR, augmented reality.

This article is a narrative review rather than a systematic review. Literature was identified through targeted searches of PubMed/MEDLINE, Scopus, and Google Scholar, last updated on 8 August 2026. Search concepts combined “artificial intelligence”, “machine learning”, “deep learning”, “computer vision”, and “large language models” with “cardiac surgery”, “cardiothoracic surgery”, “perioperative care”, “surgical training”, and “surgical education”; targeted searches also addressed cardiovascular imaging, simulation, governance, regulation, data protection, and cybersecurity. The article synthesizes peer-reviewed literature on AI in cardiac surgery, perioperative medicine, surgical data science, and surgical education, with priority given to systematic reviews, meta-analyses, clinically relevant studies, and regulatory or professional sources. Because cardiac-specific evidence on AI-supported education and intraoperative computer vision remains limited, selected evidence from general, laparoscopic, urological, and robotic surgery is included where the underlying educational or technical principle is transferable. Those boundaries are stated explicitly so that findings from other specialties are not presented as established cardiac-surgical evidence. No PRISMA process or formal meta-analysis was undertaken. The principal clinical, educational, and governance domains considered in this review are summarized in Figure 1.

## 3. AI Across the Cardiac Surgical Pathway

### 3.1. Preoperative Assessment and Planning

Risk prediction is currently the most developed application of machine learning in cardiac surgery. Models have been trained to estimate mortality, acute kidney injury, prolonged ventilation, bleeding, intensive care length of stay, readmission, and other adverse outcomes. Systematic reviews describe a rapidly expanding literature, but they also identify recurrent limitations: single-centre datasets, retrospective design, inconsistent outcome definitions, incomplete reporting, inadequate external validation, and a focus on statistical performance rather than clinical utility [4,5,6,7]. In a meta-analysis of mortality prediction after cardiac surgery, machine learning models did not demonstrate a statistically significant advantage over logistic regression when discrimination was compared using the C-index [7]. This finding does not imply that machine learning lacks value. It does, however, argue against assuming superiority merely because a model is more complex.

The most promising gains may arise when conventional clinical variables are combined with high-dimensional data that are difficult to incorporate into traditional scores. Deep learning systems can extract latent features from electrocardiograms and imaging studies, and AI-enabled electrocardiography has identified atrial fibrillation signatures during sinus rhythm and classified rhythm disturbances with high accuracy in large datasets [8,9]. In medical imaging, deep learning has achieved strong performance in segmentation, classification, and feature extraction [10]. For cardiac surgery, these methods could support anatomical mapping, assessment of aortic calcification, ventricular and valvular characterization, conduit planning, or prediction of procedural complexity. Their clinical contribution will depend on whether they improve decisions beyond expert interpretation and established imaging workflows.

A clinically useful model must do more than predict accurately. It should address a clearly defined decision, use data available at the relevant time, remain calibrated across institutions and patient groups, and provide information that can alter management. External validation is essential, but local validation is equally important because referral patterns, operative strategies, perioperative pathways, and data quality vary substantially between cardiac-surgical programmes. Calibration drift should be anticipated rather than treated as an exceptional event.

Cardiovascular imaging is also a natural interface between cardiac surgeons, cardiologists, and imaging specialists. AI-derived biomarkers from coronary computed tomography angiography can characterize plaque and pericoronary adipose-tissue inflammation, while automated echocardiographic and cardiovascular magnetic resonance analysis can support quantification, phenotyping, and integration of imaging with clinical data [11,12,13]. In the surgical pathway, CT-based segmentation and reconstruction may improve anatomical mapping and procedural planning. These outputs should be interpreted by the multidisciplinary heart team, because an imaging biomarker or automated contour becomes clinically useful only when it changes patient selection, timing, or operative strategy beyond expert review.

### 3.2. Intraoperative Support

Robotic cardiac surgery is often discussed together with AI, although the two are not synonymous. Current robotic platforms primarily provide telemanipulation, motion scaling, tremor reduction, enhanced visualization, and ergonomic advantages; these functions do not by themselves constitute autonomous intelligence. The evolution of robotic cardiac surgery nevertheless creates a technical platform on which AI-enabled perception, workflow recognition, and decision support may be developed [14]. Maintaining this distinction is important because attributing the established benefits of robotic systems to AI overstates the present evidence.

Intraoperative AI research has focused largely on computer vision. Surgical video can be analysed to identify instruments, anatomical structures, procedural phases, and deviations from an expected workflow. Surgical data science aims to integrate video, device data, physiological signals, and contextual information into systems that support safer and more reproducible interventions [15,16]. In laparoscopic cholecystectomy, for example, semantic-segmentation models have been developed to identify anatomical structures and potentially support intraoperative guidance [17]. These studies demonstrate technical feasibility, but they are not evidence that equivalent systems are ready for cardiac surgery.

Cardiac procedures present additional challenges: blood and smoke may obscure the field; tissues deform continuously; cardiopulmonary bypass changes physiology; operations vary according to anatomy and surgeon strategy; and rare but catastrophic events may have few examples available for model training. A useful intraoperative system must operate with low latency, recognize when image quality or context is outside its competence, and fail safely. It should not generate an authoritative-looking recommendation when uncertainty is high. At present, the concept of an AI “co-pilot” is credible as a research direction, but real-time autonomous guidance in cardiac surgery remains investigational.

### 3.3. Postoperative Care and Workflow

Postoperative care generates dense time-series data that may be well suited to dynamic risk modelling. Continuous laboratory, haemodynamic, ventilatory, and nursing data could be used to update the probability of complications as the patient’s condition evolves. This may be more clinically useful than a single preoperative estimate, particularly for early detection of bleeding, low-output syndrome, renal injury, respiratory failure, infection, or prolonged intensive care requirements [5,6]. Yet, prediction alone is insufficient. Alert timing, threshold selection, workflow integration, and the response expected from clinicians must be specified prospectively. A model that produces frequent non-actionable alerts may increase cognitive burden rather than improve care.

Natural-language processing and ambient documentation tools may reduce clerical work by drafting notes, summarizing records, or extracting structured data. Early co-design research has explored the requirements for AI documentation assistants, while the association between clerical burden and professional burnout is well established [18,19]. These tools should be regarded as drafting systems, not independent authors of the medical record. Operative details, diagnoses, medication changes, and discharge instructions require clinician verification, because fluent language can conceal factual errors or omissions.

AI may also assist scheduling, operating-room utilization, intensive care bed planning, and supply management. These are potentially high-value applications because they affect the reliability of the entire service. However, operational models can reproduce historical inequities if they are trained on resource allocation patterns rather than clinical need. Their optimization target must therefore be explicit: reducing delay is not equivalent to maximizing patient benefit, and efficiency should not be achieved by systematically disadvantaging complex or vulnerable patients (Table 1).

## 4. AI in Cardiothoracic Surgical Education

### 4.1. Simulation and Deliberate Practice

Simulation has long provided a way to practice technical tasks without exposing patients to the early stages of a learning curve. Randomized evidence predating the current AI era showed that virtual-reality training could improve performance in the operating room, and proficiency-based simulator training has translated to better laparoscopic suturing performance [21,22]. AI can extend this educational model by adapting task difficulty, detecting recurrent errors, comparing performance with expert benchmarks, and generating targeted feedback. The educational value lies not in simulation alone but in deliberate practice: repeated performance of a defined task, immediate feedback, correction, and reassessment.

For cardiac surgery, simulation may be particularly useful for cannulation, coronary anastomosis, valve repair steps, endoscopic instrument handling, robotic console skills, cardiopulmonary bypass emergencies, and conversion to open surgery. Rare events are especially important. A trainee may complete formal training without encountering certain catastrophic complications often enough to develop a reliable response. Simulation can make such exposure intentional rather than accidental.

The limitation is fidelity of consequence. A simulator can reproduce anatomy or instrument movement, but it does not fully reproduce the emotional pressure, team dynamics, tissue variability, or ethical responsibility of operating on a patient. AI-guided simulation should therefore complement graded operative responsibility and structured debriefing, not replace them.

### 4.2. Objective Assessment of Technical Skill

Assessment of surgical skill has traditionally depended on expert observation and structured rating scales. The Objective Structured Assessment of Technical Skills established a framework for more reliable evaluation, but it remains resource-intensive and subject to observer variation [23]. Computer vision and motion analysis can quantify instrument trajectories, economy of movement, gesture sequence, procedural time, and error patterns. Deep learning models have classified surgical actions and estimated performance level from video, while systematic reviews show a large and rapidly growing body of automated and AI-based assessment tools [24,25].

The attraction is clear: automated systems could provide frequent formative feedback, identify specific components of a task that need improvement, and support longitudinal tracking. They may also reduce institutional variation in assessment. Nonetheless, many systems have been evaluated on small datasets, standardized simulator tasks, or recordings from a limited number of surgeons. High classification accuracy in a laboratory does not establish validity for credentialing. Before an automated score is used for progression or certification, evidence is required that the score reflects clinically meaningful competence, is robust across procedures and institutions, and is not confounded by equipment, camera angle, body habitus, or preferred operative style.

Cardiothoracic-specific evidence is beginning to emerge. A 2026 feasibility study used AI-based hand tracking during suturing and knot-tying tasks and found that motion-derived variables improved prediction of experience when combined with procedural time and conventional assessment [29]. This is a useful proof of concept, but it should not be interpreted as a validated credentialing system. The appropriate near-term role is formative: augmenting faculty feedback and helping trainees understand how their movement patterns change with practice.

### 4.3. Generative AI and Cognitive Training

Large language models can generate explanations, clinical questions, case variants, checklists, and structured feedback. They may help trainees rehearse differential diagnosis, operative planning, consent discussions, and postoperative communication. Their accessibility is a genuine educational advantage. Surgical-education reviews nevertheless show that implementation evidence remains limited and that most AI interventions have concentrated on technical skill assessment in simulation rather than patient-level outcomes [26,27,28]. Large language models also produce variable results and may state incorrect information with persuasive confidence [30].

The safest educational use is supervised and source-grounded. Trainees should be taught to verify claims against guidelines and primary literature, to recognize uncertainty, and to document when an AI system materially influenced an educational product. A model can help generate cases or explanations; it cannot determine whether a trainee has understood the pathophysiology, appreciates the limits of the evidence, or will make a safe decision when the case departs from the expected pattern.

## 5. Risks of Over-Reliance

### 5.1. Automation Bias and Cognitive Off-Loading

Automation bias occurs when users favour a system recommendation despite contradictory information or fail to act because the system did not issue an alert. In surgery, this risk is amplified by time pressure and by the apparent precision of numerical outputs. A probability displayed to two decimal places may appear more certain than the underlying data justify. Over time, repeated acceptance of automated recommendations can shift the surgeon’s role from active reasoning to passive confirmation.

The concern is not that every use of AI causes deskilling. Decision support can reduce omission and help clinicians process information that would otherwise be inaccessible. The risk arises when the system replaces rather than scaffolds reasoning. If a trainee receives continuous step-by-step prompting, the procedure may be completed successfully without a durable mental model of anatomy, sequencing, and failure management. The critical educational test is whether performance persists when guidance is removed.

### 5.2. Manual Skill, Adaptability, and Failure Management

Cardiac surgeons must remain competent when imaging is incomplete, robotic access is lost, a device malfunctions, bleeding obscures the field, or conversion is required. These situations cannot be managed by following the normal workflow more accurately; they require deviation from it. AI systems trained predominantly on routine procedures may perform least reliably in precisely the situations in which expert judgement matters most.

Evidence of AI-induced manual skill erosion in cardiac surgery is not yet established, and the claim should not be presented as a demonstrated outcome. It is, however, a plausible training hazard supported by broader concerns about cognitive off-loading and unintended consequences of clinical machine learning [31,32,33]. Programmes should therefore measure unaided competence explicitly. Simulation should include loss of navigation, corrupted data, incorrect prompts, device failure, emergency conversion, and disagreement between the algorithm and the operative findings.

### 5.3. Mentorship and Professional Formation

Surgical apprenticeship transmits more than a sequence of manoeuvres. Through observation, discussion, and shared responsibility, trainees learn how experienced surgeons frame uncertainty, recover from error, communicate with the team, and decide when not to operate. These forms of tacit knowledge are difficult to encode. An automated tutor may provide immediate and consistent feedback, but it does not carry responsibility for the patient or understand the institutional and interpersonal context of a decision.

AI should therefore reduce low-value educational workload while protecting high-value human interaction. Automated metrics can identify moments for review; faculty should interpret those moments with the trainee. A video-analysis system might flag excessive instrument travel or an unusual sequence, but the mentor remains responsible for determining whether that pattern reflects poor technique, an anatomical constraint, or an appropriate adaptation.

### 5.4. Model Opacity, Dataset Shift, and Bias

Complex models may provide limited explanation for an individual prediction. Even when an explanation method is available, it may describe model behaviour rather than causal clinical reasoning. This matters in cardiac surgery because model inputs may encode institutional practice, referral pathways, or treatment decisions rather than underlying disease. Performance can deteriorate when the patient population, imaging protocol, device, documentation system, or clinical pathway changes.

Medical AI studies have repeatedly shown that limited external validation and dataset weaknesses can produce exaggerated performance estimates [32,34]. A widely deployed proprietary sepsis model performed substantially worse on external evaluation than reported during development [35]. Algorithmic bias may also arise when healthcare expenditure or access is used as a proxy for clinical need, thereby reproducing racial and socioeconomic inequities [36]. Responsible deployment requires representative data, subgroup analysis, prospective monitoring, and a mechanism to suspend use when performance deteriorates [37,38].

### 5.5. Accountability, Cost, and Institutional Dependence

An AI recommendation does not transfer professional responsibility away from the clinical team. Before deployment, institutions should define who reviews model performance, who responds to an alert, how disagreements are documented, and how adverse events involving the system are investigated. Vendors should provide sufficient information about intended use, training data, updates, and known limitations for safe local governance.

Cost deserves similar attention. New technology can increase expenditure through acquisition, maintenance, integration, training, and changes in case selection, even when individual procedures become more efficient [39]. Dependence on a proprietary platform may also reduce institutional resilience. A cardiac-surgical programme should be able to maintain safe care during downtime and should avoid allowing essential expertise to become inseparable from a single commercial system.

## 6. Safeguarding Surgical Standards: A Human-in-Command Framework

The appropriate governance model is human-in-command rather than merely human-in-the-loop. A clinician who is required to click “accept” but lacks the time, information, or authority to challenge a system is not exercising meaningful oversight. Human-in-command means that the clinical team retains the competence and operational capacity to understand, question, override, and, when necessary, continue safely without the technology (Table 2).

First, every AI system should have a clinically meaningful intended use and a validation plan. Statistical performance should be reported alongside calibration, clinical utility, subgroup performance, and comparison with existing practice. Second, programmes should preserve unaided competence. Case logs alone are insufficient; periodic assessment should include manual technique, open conversion, crisis management, and decision-making without algorithmic support. Third, failure-mode training should be routine. Trainees must experience incorrect outputs and system unavailability in simulation so that skepticism and recovery are practiced rather than assumed. Fourth, automated assessment should initially be formative. High-stakes use requires evidence of validity, reliability, fairness, and consequences across institutions. Fifth, mentorship should be redesigned rather than reduced. AI can identify learning opportunities and reduce repetitive scoring, allowing faculty to focus on judgement, strategy, and professional development. Sixth, governance should include surgeons, anaesthesiologists, perfusionists, nurses, educators, data scientists, information-security specialists, patients, and institutional leadership. The U.S. Food and Drug Administration has emphasized lifecycle oversight for AI/ML-based medical software, and the American College of Surgeons has developed educational resources addressing clinical, performance, and ethical aspects of AI [40,41].

European deployment must also be considered within complementary frameworks, including the phased implementation of the European Union Artificial Intelligence Act and the Medical Device Regulation. AI software that is a medical device or a safety component may fall within the high-risk framework, with requirements concerning risk management, data governance, technical documentation, human oversight, and post-market monitoring [42,43]. Operative video, imaging, and trainee-performance data require a defined lawful basis, data minimization, role-based access, retention limits, and safeguards for secondary use under the General Data Protection Regulation. Cybersecurity governance should include secure interfaces, access logging, update control, incident response, and resilience against model or data compromise [44,45]. Institutional policies and consent processes should address both patient and staff or trainee data, including whether recordings may be used for assessment or model development.

Finally, institutions should monitor whether AI changes behaviour in unintended ways. Relevant outcomes include not only morbidity, mortality, and efficiency but also alert burden, override patterns, near misses, trainee independence, manual skill retention, case distribution, and disparities between patient groups. A system that improves an intermediate metric while weakening team resilience should not be considered successful.

## 7. Clinical Implementation and Future Perspectives

Translation into routine care will depend as much on implementation as on model performance. Regulatory approval is necessary but not sufficient: systems must interoperate with electronic health records, imaging archives, operating-room devices, and monitoring platforms without adding duplicate data entry or alert burden. Clinician acceptance will depend on transparent intended use, workflow co-design, training, rapid access to human review, and evidence that benefits persist in local practice. Cost-effectiveness studies should include acquisition, integration, cybersecurity, updates, staff time, downtime, and the opportunity cost of displaced workflows, not only per-case technical efficiency [38,39].

Over the next decade, multimodal foundation models may combine clinical history, imaging, waveforms, laboratory data, operative video, and text to support longitudinal prediction and context-aware decision support. Vision–language models already demonstrate broad task transfer in echocardiography, but their ability to improve cardiac-surgical decisions and outcomes remains unproven [20]. Patient-specific digital twins may link anatomical models with physiology and computational simulation to test procedural scenarios before an operation; for now, these systems are research platforms rather than substitutes for Heart Team judgement [46].

Autonomous image interpretation and integrated decision-support platforms may eventually connect preoperative imaging, intraoperative events, and postoperative trajectories. The appropriate endpoint is not unqualified autonomy, but calibrated assistance that declares uncertainty, identifies out-of-distribution inputs, preserves an auditable record, and escalates to human expertise. Prospective, multicentre studies should evaluate patient outcomes, equity, team behaviour, skill retention, interoperability, and cost-effectiveness before such systems are adopted at scale.

## 8. Conclusions

AI has credible and potentially important roles in cardiac surgery, particularly in risk prediction, data integration, workflow support, and objective analysis of technical performance. The evidence is strongest for prediction and technical feasibility; it is weaker for prospective clinical benefit, routine intraoperative guidance, and cardiac-specific educational outcomes. Claims should therefore remain proportional to the data.

The principal educational opportunity is not instruction without teachers but better use of teachers and practice. AI can make feedback more frequent, identify patterns that are difficult to observe, and create structured opportunities to rehearse uncommon events. Its value will be lost if those advantages are purchased by reducing operative responsibility, mentorship, or the capacity to act independently.

Cardiac surgery should adopt AI with the same discipline applied to any high-risk technology: a defined indication, evidence of benefit, transparent limitations, competent users, failure planning, and continuous surveillance. The goal is not to produce surgeons who perform well only when supported by an algorithm. It is to develop surgeons whose judgement and technical skill are strengthened by AI and who remain accountable for its use and dependable when it fails.

## Figures and Tables

**Table 1:** Maturity of evidence for principal AI applications relevant to cardiac surgery.

Application	Evidence Base in Cardiac Surgery	Evidence Maturity	Practical Interpretation
Preoperative and perioperative risk prediction	Multiple cardiac-specific systematic reviews and a meta-analysis; external validation and clinical-utility evidence remain inconsistent [4,5,6,7]	Established evidence base; implementation emerging	The most mature domain, but incremental benefit and routine deployment remain limited
AI-enabled ECG and cardiovascular imaging	Large cardiac imaging studies and reviews; limited evidence that AI-derived findings improve surgical decisions or outcomes [8,9,10,11,12,13,20]	Emerging	Supports biomarker extraction, phenotyping, and planning; Heart Team interpretation remains essential
Postoperative monitoring and workflow	Cardiac and perioperative retrospective models; prospective workflow and outcome evidence remain limited [5,6,18,19]	Emerging	Requires prospective evaluation, actionable alerts, and integration with existing systems
Intraoperative computer vision, navigation, and decision support	Predominantly non-cardiac technical-feasibility and surgical data-science studies [15,16,17]	Investigational	Cardiac use should remain supervised research rather than autonomous guidance
Simulation and automated skill assessment	Mostly laparoscopic, urological, and robotic studies, with one cardiothoracic feasibility study [21,22,23,24,25,26,27,28,29]	Investigational in cardiac surgery	Appropriate for supervised formative feedback; not established for high-stakes credentialing
Generative AI, documentation, and cognitive support	Early non-cardiac implementation and surgical education literature [18,26,27,28,30]	Investigational	Drafting and cognitive support require clinician or faculty verification

**Table 2:** Practical safeguards for AI integration in cardiac surgery and training.

Domain	Principal Risk	Recommended Safeguard	Educational Implication
Intended use	Use outside the problem for which the model was developed	Define the decision, population, timing, users, and prohibited uses	Teach indication and limitation, not just operation of the tool
Validation	Optimistic local or retrospective performance	Independent external validation, local testing, calibration assessment, and subgroup analysis	Trainees should understand discrimination, calibration, and generalizability
Human oversight	Automation bias and passive confirmation	Display uncertainty; require meaningful review; preserve authority to override	Assess reasoning when the AI recommendation is absent or intentionally incorrect
Core competence	Loss of manual, open, or crisis-management skill	Maintain case exposure, simulation, and periodic unaided competency assessment	Include technology failure, emergency conversion, and non-standard anatomy
Mentorship	Replacement of feedback by automated scores	Use AI to identify learning events; retain faculty interpretation and debriefing	Automated feedback remains formative unless validity for high-stakes use is established
Monitoring	Dataset shift and performance degradation	Continuous audit, incident reporting, update control, and stop criteria	Teach that validation is a continuing process, not a one-time approval
Accountability	Unclear responsibility among surgeon, institution, and vendor	Predefine roles, documentation, escalation, and adverse-event review	Professional responsibility remains with appropriately trained clinicians and institutions

## Data Availability

No new data were created or analyzed in this study. Data sharing is not applicable to this article.
